# Manipulation of Surface Potential Distribution Enhances Osteogenesis by Promoting Pro‐Angiogenic Macrophage Polarization via Activation of the PI3K‐Akt Signaling Pathway

**DOI:** 10.1002/advs.202414278

**Published:** 2024-12-30

**Authors:** Qun Cui, Xiaona Zheng, Yunyang Bai, Yaru Guo, Shuo Liu, Yanhui Lu, Lulu Liu, Jia Song, Yang Liu, Boon Chin Heng, Fuping You, Mingming Xu, Xuliang Deng, Xuehui Zhang

**Affiliations:** ^1^ Department of Dental Materials & Dental Medical Devices Testing Center Peking University School and Hospital of Stomatology Beijing 100081 P. R. China; ^2^ Oral Translational Medicine Research Center Joint Training base for Shanxi Provincial Key Laboratory in Oral and Maxillofacial Repair Reconstruction and Regeneration The First People's Hospital of Jinzhong Jinzhong Shanxi 030600 P. R. China; ^3^ National Engineering Research Center of Oral Biomaterials and Digital Medical Devices NMPA Key Laboratory for Dental Materials Beijing Laboratory of Biomedical Materials & Beijing Key Laboratory of Digital Stomatology Peking University School and Hospital of Stomatology Beijing 100081 P. R. China; ^4^ Department of Geriatric Dentistry Peking University School and Hospital of Stomatology Beijing 100081 P. R. China; ^5^ Institute of Systems Biomedicine School of Basic Medical Sciences NHC Key Laboratory of Medical Immunology Beijing Key Laboratory of Tumor Systems Biology Peking University Health Science Center Beijing 100191 P. R. China

**Keywords:** angiogenesis, bone regeneration, heterogeneous surface potential distribution, PI3K‐Akt signaling pathway, pro‐angiogenic macrophage polarization

## Abstract

Regulation of the immune response is key to promoting bone regeneration by electroactive biomaterials. However, how electrical signals at the micro‐ and nanoscale regulate the immune response and subsequent angiogenesis during bone regeneration remains to be elucidated. Here, the distinctly different surface potential distributions on charged poly(vinylidene fluoridetrifluoroethylene) (P(VDF‐TrFE)) matrix surfaces are established by altering the dimensions of ferroelectric nanofillers from 0D BaTiO_3_ nanoparticles (homogeneous surface potential distribution, HOPD) to 1D BaTiO_3_ nanofibers (heterogeneous surface potential distribution, HEPD). Compared to HOPD, HEPD is significantly better at inducing the M2 polarization of macrophages and promoting neovascularization, which results in accelerated bone regeneration in vivo. The transcriptomic analysis reveals that macrophages modulated by HEPD display high expression levels of pro‐angiogenic genes, which is corroborated by tube‐formation assays, RT‐qPCR, and western blot analyses in vitro. Mechanistic explorations elucidate activation of the PI3K‐Akt signaling pathway, which in turn induces the polarization of macrophages toward a pro‐angiogenic phenotype. This study elucidates the cascade of biological processes by which heterogeneous electrical signals at the micro‐ and nanoscale modulate macrophage functions to promote vascularization and bone regeneration. Hence, this study provides new insights into how the micro‐ and nanoscale distribution characteristics of electrical signals facilitate bone regeneration.

## Graphical abstract

The polarized BaTiO_3_ nanofibers (BTNF)/P(VDF‐TrFE) nanocomposite membrane provides heterogeneous surface potential distribution within the bone repair microenvironment, which induces macrophage differentiation toward the M2 phenotype and activates the PI3K‐Akt signaling pathway in macrophages to enhance their pro‐angiogenic functions, thereby enhancing angiogenic differentiation of endothelial cells. Ultimately, this cascade of biological processes accelerates bone defect repair.

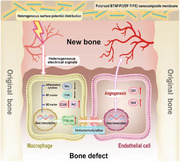

## 1 Introduction

The physicochemical characteristics of biomaterials, including chemical composition, structure, stiffness, electrical properties, wettability, and pH, are pivotal for modulating cell behavior and maintaining tissue homeostasis.^[^ ^1^, ^2^, ^3^, ^4^, ^5^, ^6^ ^]^ Among the diverse array of biomaterials, electroactive biomaterials are particularly effective in enhancing bone regeneration via biomimetic recapitulation of the normal electrical microenvironment within the bone defect area.^[^ ^2^, ^7^, ^8^ ^]^ The electrical microenvironment where cells reside, is heterogeneous at the micro‐ and nanoscale.^[^ ^9^, ^10^, ^11^, ^12^, ^13^ ^]^ This heterogeneity plays a crucial role in cell growth, differentiation, migration, intercellular interactions, and various other cellular functions. Following this cue, we fabricated macroscopic electroactive nanocomposite membranes and further developed the electrical properties of these materials by synthesizing nanocomposite membranes with heterogeneous surface potential distribution (HEPD) at the micro‐ and nanoscale.^[^ ^10^ ^]^ This innovation mimics the heterogeneous electrical microenvironment at the micro‐ and nanoscale within the bone regeneration microenvironment. Compared to homogeneous electrical signals, our nanocomposite membranes with HEPD were significantly better at promoting the osteogenic differentiation and mineralization of mesenchymal stem cells by enhancing cellular mechanosensitivity, thereby improving the efficacy of bone repair with electroactive materials.^[^ ^10^ ^]^ However, how heterogeneous electrical signals at the micro‐ and nanoscale modulate the cascade of biological processes including immune response and subsequent angiogenesis during bone regeneration remains to be elucidated.

Studies have demonstrated that immune cells can produce and release a variety of pro‐angiogenic factors, to create a microenvironment favorable to angiogenesis thereby accelerating bone regeneration.^[^ ^14^ ^]^ As the major immune cells that form the first line of defense in the innate immune system, macrophages are found in almost all tissues and play a crucial role in tissue repair.^[^ ^15^, ^16^, ^17^ ^]^ In particular, macrophages have garnered extensive attention due to their high plasticity in displaying various phenotypes, with the M1 phenotype exhibiting pro‐inflammatory functions, while the M2 phenotype contributes to the regression of inflammation, promotion of tissue repair, and maintenance of tissue homeostasis.^[^ ^16^, ^18^, ^19^, ^20^, ^21^, ^22^, ^23^ ^]^ Previous studies have confirmed that electroactive biomaterials can effectively regulate the polarization of macrophages to the M2 phenotype, thereby promoting the regeneration of blood vessels that ultimately facilitate the repair of bone defects.^[^ ^24^, ^25^, ^26^ ^]^ However, the majority of published research studies on electroactive biomaterials for bone repair have focused on the regulation of immune response and vascular regeneration by macroscopic homogeneous electrical signals. There is still a lack of investigations on the cascade of biological processes involved in regulating the immune response, promoting vascular regeneration, and ultimately facilitating bone regeneration by heterogeneous distributions of electrical signals at the micro‐ and nanoscale. This poses a major obstacle in precisely further enhancing the biological performance of electroactive biomaterials.

**Scheme 1:**
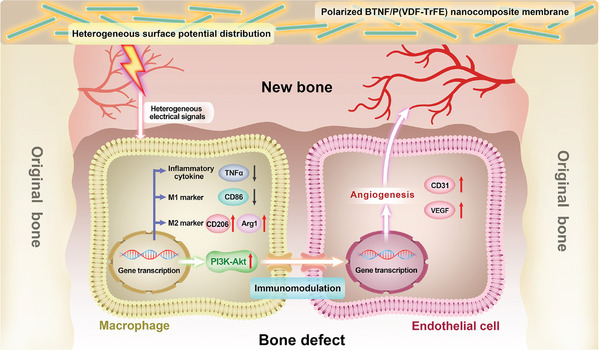
Schematic illustration of HEPD enhancing osteogenesis by promoting M2 and pro‐angiogenic polarization of macrophages that facilitate angiogenesis. When the polarized BTNF/P(VDF‐TrFE) nanocomposite membrane covered the bone defect, the HEPD provided by the membrane downregulates the expression of the M1 marker CD86 and inflammatory cytokine TNFα in macrophages, and further induces their polarization toward the M2 phenotype, thus establishing an anti‐inflammatory microenvironment. Meanwhile, the HEPD also activates the PI3K‐Akt signaling pathway in macrophages to enhance their pro‐angiogenic functions, thereby enhancing subsequent angiogenesis of endothelial cells. Ultimately, this cascade of biological processes accelerates bone defect repair.

In this study, we employed ferroelectric nanocomposite membranes specifically designed to provide HEPD, which incorporated optimized BaTiO_3_ nanofibers (BTNF) within poly(vinylidene fluoridetrifluoroethylene) (P(VDF‐TrFE)) matrix.^[^ ^10^ ^]^ This facilitated our investigations of the regulatory roles of micro‐ and nanoscale heterogeneous electrical signals in immune responses and vascular regeneration during bone regeneration. By combining vascular microfil‐perfusion and immunofluorescence staining, we demonstrated that HEPD can promote angiogenesis and subsequent bone regeneration in vivo. Meanwhile, it was also observed that HEPD could better promote the M2 polarization of macrophages both in vitro and in vivo, as compared to homogeneous surface potential distribution (HOPD). Gene ontology (GO) analysis of differentially expressed genes (DEGs) revealed that genes highly expressed in macrophages modulated by HEPD were significantly enriched in the function associated with “angiogenesis”. Consistently, the tube‐formation assay, RT‐qPCR, and western blot analyses showed that macrophages modulated by HEPD could enhance the angiogenic function of human umbilical vein endothelial cells (HUVECs) in vitro. Mechanistically, HEPD promoted the pro‐angiogenic functions of macrophages by activating the PI3K‐Akt signaling pathway. In summary, this study thus established the relationship between micro‐ and nanoscale heterogeneous electrical signals, pro‐angiogenic macrophages, angiogenesis, and bone regeneration, thereby facilitating a better understanding of the underlying mechanisms by which micro‐ and nanoscale heterogeneous electrical signal distribution regulate bone regeneration (**Scheme** **1**).

## 2 Results and Discussion

### 2.1 The Establishment of Heterogeneous Surface Potential Distribution (HEPD) on Ferroelectric Nanocomposite Membranes

**Figure 1:**
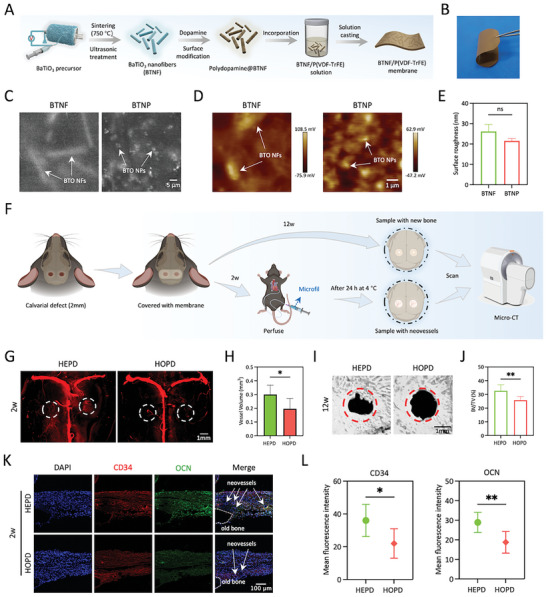
HEPD enhances angiogenesis and subsequent bone regeneration in vivo. A) Schematic diagram of the fabrication process of BTNF nanocomposite membranes. B) Image of a BTNF nanocomposite membrane. C) Representative SEM images of BTNF and BTNP nanocomposite membranes. The white arrows denote the BaTiO_3_ nanofibers (BTO NFs) and BaTiO_3_ nanoparticles (BTO NPs). Scale bars: 5 µm. D) Surface potentials of BTNF and BTNP nanocomposite membranes. Scale bars: 1 µm. E) Roughness of BTNF and BTNP nanocomposite membranes, *n* = 3. F) Schematic illustration of angiogenesis and bone regeneration assessment. G) Representative micro‐CT images of vasculature within the microfil‐perfused craniums showed that compared with the HOPD group, neovessel formation was significantly enhanced in the HEPD group. The dashed lines indicate bone defect areas. Scale bars: 1 mm. H) Quantitative analysis of neovessel volumes in G showed increased vessel volume in the HEPD group, *n* = 6. I) Representative micro‐CT images of mice with 2mm‐diameter calvarial full‐thickness defects at 12 weeks post‐surgery. The red dotted lines denote the boundary between the nascent bone and the host bone. Scale bars: 1 mm. J) Quantitative analysis of bone volume fraction (BV/TV), *n* = 6. K) Representative immunofluorescence staining images of regenerated tissues at 2 weeks post‐surgery. The dashed lines denote the indicated tissues. Scale bars: 100 µm. L) Analysis of mean fluorescence intensities representing expression of CD34 and osteocalcin (OCN) in K, *n* = 6. Statistical significance was assessed using the unpaired Student's t‐test. ns, not significant; *p < 0.05; **p < 0.01.

Our previous study has demonstrated that BTNF obtained through electrospinning, after sintering at 750 °C for 6 h and being subjected to ultrasonic treatment for 5 min, could achieve an optimal aspect ratio. This results in a heterogeneous surface electric potential distribution on the charged nanocomposite membranes after incorporating optimized BTNF within the P(VDF‐TrFE) matrix, which facilitated cell recognition, adhesion, and osteogenic differentiation.^[^ ^10^ ^]^ Based on this, we fabricated flexible electroactive nanocomposite membranes consisting of dopamine‐modified BaTiO_3_ nanoparticles (BTNP) or BTNF as fillers and P(VDF‐TrFE) as the matrix via the solution casting method (**Figure** **1**A,B). The two membranes were subjected to corona discharge at room temperature to obtain polarized electroactive nanocomposite membranes that provided HOPD and HEPD at the micro‐ and nanoscale, respectively, thus facilitating the subsequent investigation of differences in biological effects between them. The Scanning Electron Microscopy (SEM) images revealed that BTNF was anisotropically distributed within the polymer matrix, while BTNP was isotropically homogeneous (Figure 1C). The surface electric potential distribution was further characterized by Scanning Probe Microscopy (SPM) imaging (Figure 1D). It was observed that the surface electric potential of the BTNF nanocomposite membrane exhibited a line‐like anisotropic distribution, while the BTNP nanocomposite membrane displayed a homogeneous high‐potential pitting distribution. This difference might be due to the increase in local surface electric potential caused by the different shapes of BaTiO_3_ fillers after polarization.^[^ ^10^ ^]^ However, there was no significant difference in the roughness between the two membranes (Figure 1E). Additionally, the electrical properties of the membranes were characterized, revealing that the piezoelectric constant *d_33_*, the polar 𝛽‐phase content and the polarization‐electric field (P‐E) loop were similar between the two membranes (Figure S1A–C, Supporting Information). Based on these findings, we confirmed that BTNF and BTNP nanocomposite membranes differed in the surface electric potential distribution at the micro‐ and nanoscale, with no significant differences in other general properties being observed. This conclusion was consistent with our previous work.^[^ ^10^ ^]^ Hence, there is a rational basis for utilizing BTNF nanocomposite membranes to investigate the effect of micro‐ and nanoscale heterogeneous electrical signals in regulating the bone repair process.

### 2.2 HEPD Enhances Angiogenesis and Subsequent Bone Regeneration In Vivo

The vascular network, distributed throughout bone tissue, plays a crucial role in bone development and regeneration by delivering oxygen, essential nutrients, cells, and regenerative factors.^[^ ^27^, ^28^ ^]^ Following this cue, we first investigated whether HEPD could promote angiogenesis during bone regeneration (Figure 1F). We constructed a mouse calvarial defect model which was covered with either polarized BTNF (HEPD group) or BTNP (HOPD group) nanocomposite membranes. At 2 weeks post‐surgery, micro‐CT angiography showed that BTNF nanocomposite membranes significantly promoted the formation of newly regenerated capillaries within the calvarial bone defect area (Figure 1G). Quantitative analysis showed a significantly increased volume of the newly formed capillaries in the HEPD group, with the volume being ≈1.5 times that of the HOPD group (Figure 1H). At 12 weeks post‐surgery, micro‐CT analysis showed that the HEPD group exhibited a significantly greater degree of regenerated bone tissue in the bone defect area compared to the HOPD group (Figure 1I). As expected, the quantitative analysis results showed a significant increase in the BV/TV of the HEPD group, which was ≈25% in the HOPD group but >30% in the HEPD group (Figure 1J). Furthermore, the effects of HEPD on angiogenesis and osteogenesis were assessed by immunofluorescence staining for CD34 (a marker of vascular endothelial progenitor cells) and OCN (an osteogenesis‐related protein) within the regenerated tissues at 2 weeks post‐surgery (Figure 1K). Quantitative analysis revealed that the intensities of CD34 and OCN were significantly higher in the HEPD group compared to the HOPD group (Figure 1L). Together, these results suggested that HEPD further promoted angiogenesis and subsequent bone regeneration in vivo.

### 2.3 HEPD Promotes the M2 Polarization of Macrophages and the Establishment of an Anti‐Inflammatory Microenvironment In Vivo

**Figure 2:**
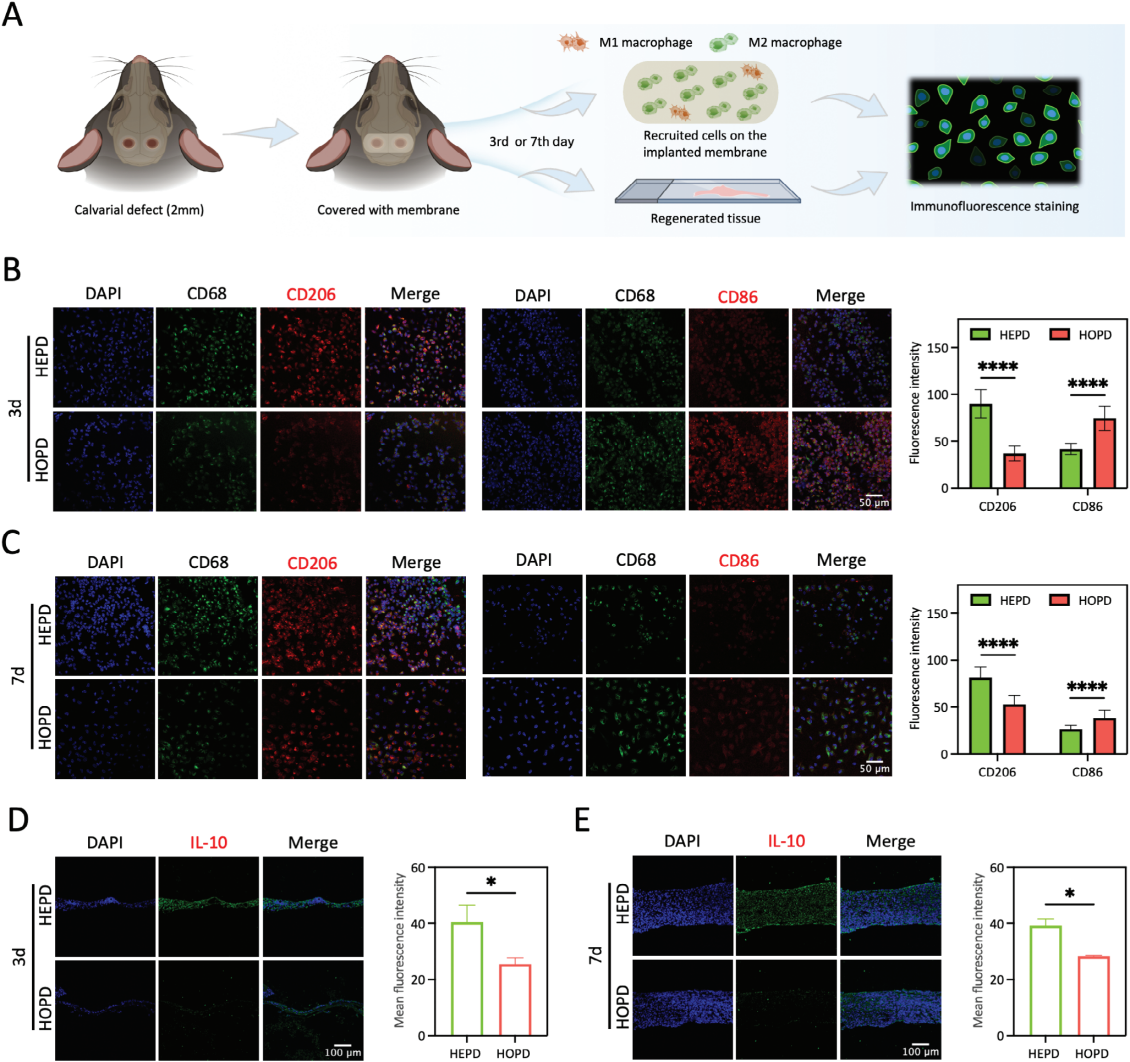
HEPD promotes the M2 polarization of macrophages and the establishment of an anti‐inflammatory microenvironment in vivo. A) Schematic illustration of the sample source for immunofluorescence staining. B,C) Representative immunofluorescence staining images and analysis of fluorescence intensity showed that the expression of the M2 macrophage surface marker CD206 was higher in the HEPD group compared to the HOPD group, while expression of the M1 macrophage surface marker CD86 was higher in the HOPD group on the 3rd and 7th day post‐surgery. Scale bars: 50 µm, *n* = 50. D,E) Representative immunofluorescence staining images and the analysis of mean fluorescence intensity showed a significant increase in IL‐10 expression within the defect area of the HEPD group compared with the HOPD group on the 3rd and 7th day post‐surgery. Scale bars: 100 µm, *n* = 3. Statistical significance was assessed using the unpaired Student's t‐test. *p < 0.05; ****p < 0.0001.

Next, we endeavored to uncover the mechanisms by which HEPD can further promote angiogenesis and osteogenesis. The immune response, particularly the polarization of macrophages, has been established as a crucial regulatory factor in vascularization during bone regeneration.^[^ ^24^, ^25^, ^26^ ^]^ Therefore, we investigated whether HEPD could modulate the polarization of macrophages, to provide a beneficial immune microenvironment conducive for tissue regeneration in vivo. Polarized BTNF or BTNP nanocomposite membranes were used to cover the calvarial defects in mice (**Figure** **2**A). On the 3rd and 7th day post‐surgery, we performed immunofluorescence staining of the recruited cells on the implanted nanocomposite membranes. We utilized CD68 and CD206 to identify M2 macrophages, while CD68 and CD86 served to label M1 macrophages.^[^ ^23^, ^29^ ^]^ As shown in Figure 2B,C, the HEPD group exhibited significantly higher expression of the M2 macrophage surface marker CD206 on the 3rd day post‐surgery, when compared to the HOPD group. In contrast, the expression of the M1 macrophage surface marker CD86 was significantly higher on the BTNP nanocomposite membranes. On the 7th day post‐surgery, the expression of CD206 increased in macrophages recruited by the BTNP nanocomposite membranes, while remaining lower than that on the BTNF nanocomposite membranes, whereas the expression of CD86 in the HOPD group was still higher. These results thus indicated that the BTNF nanocomposite membranes with HEPD promoted M2 polarization of macrophages more effectively than the BTNP nanocomposite membrane during the early stage (from day 3) of bone defect repair.

We further conducted immunofluorescence staining for detecting the expression of IL‐10, a classic anti‐inflammatory cytokine,^[^ ^30^, ^31^ ^]^ within the regenerated tissue of the bone defect area on the 3rd and 7th day post‐surgery. As expected, the results showed a significant increase in IL‐10 expression within the defect area of the HEPD group compared to the HOPD group (Figure 2D,E). Hence, these results indicated that HEPD could significantly better induce M2 polarization of macrophages in vivo, thereby providing an anti‐inflammatory microenvironment potentially beneficial for tissue repair.

### 2.4 HEPD Enhances the M2 Polarization of Macrophages In Vitro

**Figure 3:**
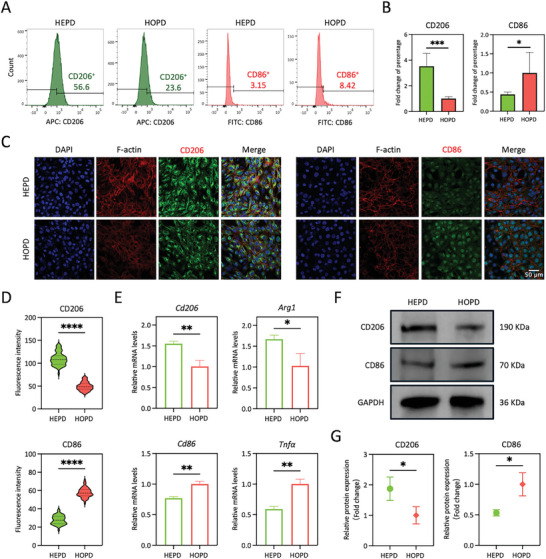
HEPD enhances the M2 polarization of macrophages in vitro. A,B) Flow cytometry showed that the expression of CD206 was significantly upregulated in the HEPD group compared with the HOPD group, whereas CD86 expression in the HEPD group was significantly downregulated, *n* = 6. C) Representative immunofluorescence staining images showed that the expression of CD206 was significantly higher while the expression of CD86 was significantly lower in the HEPD versus HOPD group. Scale bars: 50 µm. D) The immunofluorescence intensity of CD206 in the HEPD group was significantly higher while that of CD86 was significantly lower in the HEPD versus HOPD group, *n* = 50. E) RT‐qPCR showed that the gene expression levels of M2 markers *Cd206* and *Arg1* were upregulated in the HEPD group, whereas the expression levels of M1 marker *Cd86* and the inflammatory cytokine *Tnfα* were downregulated, *n* = 3. F,G) Western blot analysis showed significantly higher levels of CD206 protein expression and significantly lower levels of CD86 protein expression in the HEPD group compared with the HOPD group, *n* = 3. Statistical significance was assessed using the unpaired Student's t‐test. *p < 0.05; **p < 0.01; ***p < 0.001; ****p < 0.0001.

Subsequently, we further conducted in vitro experiments to delve into the regulatory role of HEPD on macrophage M2 polarization, thereby eliminating interference from the complex in vivo microenvironment and providing a more intuitive investigation of the regulatory effects of electrical signals on macrophages. Mouse bone marrow‐derived macrophages (BMDMs) were cultured on BTNF or BTNP nanocomposite membranes. Flow cytometry analysis revealed a significant upregulation of CD206 expression in the HEPD group, about threefold that in the HOPD group. However, the expression of CD86 in the HEPD group was significantly downregulated, about half of that in the HOPD group (**Figure** **3**A,B). Consistently, immunofluorescence staining revealed a significant difference in the expression of both CD206 and CD86 between the two groups (Figure 3C,D). Compared with the HOPD group, the fluorescence intensity of CD206 was higher in the HEPD group, while the fluorescence intensity of CD86 was lower. Additionally, RT‐qPCR analysis showed a significant upregulation of the M2 marker genes, *Cd206* and *Arg1*, in BMDMs of the HEPD group, which was accompanied by downregulation of the M1 marker gene, *Cd86*, and the inflammatory cytokine gene, *Tnfα* (Figure 3E).^[^ ^32^ ^]^ Furthermore, western blot analysis revealed significantly higher levels of CD206 protein expression and significantly lower levels of CD86 protein expression in the HEPD group compared to the HOPD group (Figure 3F,G). Hence, our results demonstrated that HEPD can better promote the M2 polarization of macrophages in vitro compared to HOPD. Together with the in vivo results, they demonstrated the regulatory role of HEPD on the immune microenvironment.

### 2.5 HEPD Promotes Angiogenesis by Regulating the Pro‐Angiogenic Functions of Macrophages

**Figure 4:**
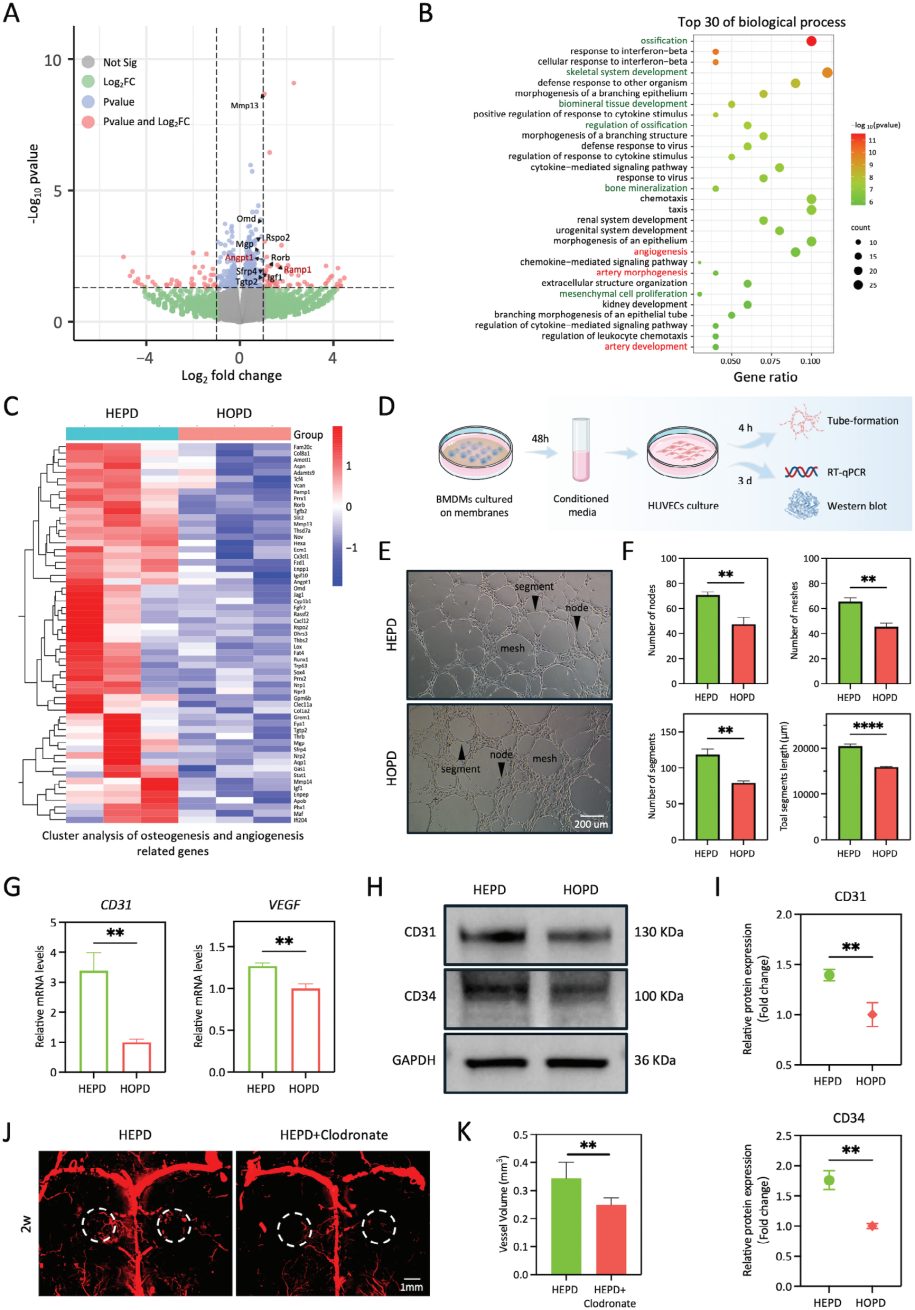
HEPD promotes angiogenesis by regulating the pro‐angiogenic functions of macrophages. A) The volcano plot of DEGs in the HEPD group compared with the HOPD group. B) The enriched GO terms of upregulated genes in the HEPD group compared with the HOPD group. The green and red highlights denote osteogenesis and angiogenesis‐related biological processes, respectively. C) Gene cluster analysis of osteogenesis and angiogenesis‐related genes in the HEPD and HOPD groups. D) Schematic illustration of angiogenic differentiation using conditioned medium from BMDMs cultured on the BTNF or BTNP nanocomposite membranes. E) Representative images of 2D tube‐formation assay of HUVECs in different conditioned mediums of BMDMs cultured on BTNF and BTNP nanocomposite membranes showed more angiogenic nodes, meshes, and segments in the HEPD versus HOPD group. Arrows denote the nodes, meshes, and segments. Scale bars: 200 µm. F) Quantitative analysis of tube‐formation in E showed an increased number of nodes, number of meshes, and the number and length of segments in the HEPD group, *n* = 3. G) RT‐qPCR showed that the gene expression levels of angiogenesis‐related genes *CD31* and *VEGF* were upregulated in the HEPD group compared with the HOPD group, *n* = 3. H,I) Western blot analysis showed significantly higher levels of angiogenesis‐related proteins CD31 and CD34 in the HEPD group compared with the HOPD group, *n* = 3. J) Representative micro‐CT images of vasculature within the microfil‐perfused craniums showed that macrophage depletion significantly reduced the capacity of HEPD to promote the formation of newly regenerated capillaries. The dashed lines indicate bone defect areas. Scale bars: 1 mm. K) Quantitative analysis of neovessel volumes in J showed decreased vessel volume in the macrophage depletion group, *n* = 6. Statistical significance was assessed using the unpaired Student's t‐test. **p < 0.01; ****p < 0.0001.

Having established that HEPD can significantly better promote vascular regeneration and M2 polarization of macrophages compared to HOPD, we hypothesized that the modulation of macrophages by HEPD may positively enhance angiogenesis. For this purpose, we collected BMDMs cultured on BTNF and BTNP nanocomposite membranes for bulk RNA‐seq analysis. As expected, DEGs analysis (**Figure** **4**A), showed that “*Ramp1*”^[^ ^33^, ^34^, ^35^ ^]^ and “*Angpt1*”^[^ ^36^, ^37^ ^]^ genes, known for their key roles in angiogenesis, were highly expressed in BMDMs of the HEPD group. The gene enrichment analysis revealed that among the top 30 biological processes highly expressed by BMDMs in the HEPD group, there were those related to osteogenesis, such as “ossification”, “skeletal system development”, and “bone mineralization”, as well as angiogenesis‐related biological processes, including “angiogenesis”, “artery morphogenesis”, and “artery development” (Figure 4B). Consistently, osteogenesis and angiogenesis‐related gene cluster analysis showed that these genes were significantly enriched in the HEPD group (Figure 4C). These results thus suggested that the BMDMs in the HEPD group display more obvious transcriptomic characteristics of promoting angiogenesis and bone regeneration than those in the HOPD group. Therefore, we hypothesized that the HEPD may play a key role in promoting angiogenesis by modulating the pro‐angiogenic functions of macrophages, ultimately promoting bone regeneration.

To validate the pro‐angiogenic functions of macrophages activated by HEPD, we performed a 2D tube‐formation assay (Figure 4D). BMDMs were cultured on either BTNF or BTNP nanocomposite membranes to obtain conditioned media. Subsequently, HUVECs were cultured in the different conditioned media for 4 h. As shown in Figure 4E,F, the number of nodes and meshes, as well as the number and length of segments, were significantly increased in the HEPD group compared to the HOPD group. We further investigated the expression of classic angiogenesis‐related genes in BMDMs in response to different electrical signals at both the mRNA and protein levels. The RT‐qPCR results showed that the expression levels of *CD31* and *VEGF* in BMDMs were significantly higher in the HEPD versus HOPD group (Figure 4G). Consistent with these results, western blot analysis also revealed significantly higher expression of CD31 and CD34 in BMDMs from the HEPD group compared to those from the HOPD group (Figure 4H,I). To further verify the crucial role of macrophages in regulating the process by which HEPD promotes angiogenesis, we constructed a mouse calvarial defect model and depleted macrophages in vivo using clodronate liposome.^[^ ^38^, ^39^, ^40^ ^]^ At 2 weeks post‐surgery, micro‐CT angiography showed that macrophage depletion significantly reduced the capacity of HEPD to promote the formation of newly regenerated capillaries (Figure 4J). The results of the quantitative analysis were consistent, and the volume of newly formed capillaries within the macrophage depletion group was significantly reduced by ≈28% when compared with the HEPD group (Figure 4K). Hence, these results indicated that HEPD could polarize macrophages toward a pro‐angiogenic phenotype, thereby enhancing their pro‐angiogenic effects on HUVECs, which in turn promoted subsequent vascular regeneration and bone repair.

### 2.6 HEPD Induces the Pro‐Angiogenic Functions of Macrophages by Activating the PI3K‐Akt Signaling Pathway

**Figure 5:**
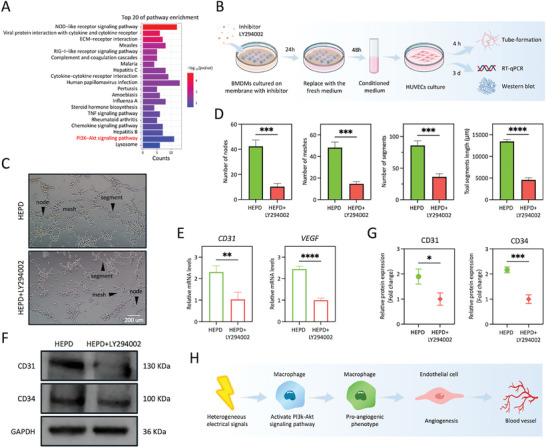
HEPD induces the pro‐angiogenic functions of macrophages by activating the PI3K‐Akt signaling pathway. A) The pathway enrichment analysis of upregulated genes in the HEPD group compared with the HOPD group. B) Schematic illustration of angiogenic differentiation using conditioned medium from BMDMs cultured on BTNF nanocomposite membranes after adding LY294002, the inhibitor of the PI3K‐Akt signaling pathway. C) Representative images of 2D tube formation assay of HUVECs in different conditioned mediums. Arrows denote the nodes, meshes, and segments. Scale bars: 200 µm. D) Quantitative analysis of tube formation in C showed decreases in the number of nodes, the number of meshes, and the number and length of segments after adding LY294002, *n* = 3. E) RT‐qPCR showed that the gene expression levels of angiogenesis‐related genes *CD31* and *VEGF* were downregulated after adding LY294002, *n* = 3. F,G) Western blot analysis showed significantly lower levels of angiogenesis‐related proteins CD31 and CD34 after adding LY294002, *n* = 3. H) Schematic illustration of the mechanisms by which heterogeneous electrical signals induced the pro‐angiogenic polarization of macrophages by activating the PI3K‐Akt signaling pathway, thereby promoting angiogenesis. Statistical significance was assessed using the unpaired Student's t‐test. *p < 0.05; **p < 0.01; ***p < 0.001; ****p < 0.0001.

To investigate the mechanisms by which HEPD modulates the pro‐angiogenic functions of macrophages, we further analyzed the bulk RNA‐seq results. The pathway enrichment analysis revealed that the genes highly expressed in BMDMs of the HEPD group were associated with the PI3K‐Akt signaling pathway, whose activation was reported to be important for angiogenesis (**Figure** **5**A).^[^ ^41^, ^42^ ^]^ This finding indicated that the HEPD on BTNF nanocomposite membranes modulated the pro‐angiogenic functions of macrophages, which might be related to the activation of the PI3K‐Akt signaling pathway.

To verify whether HEPD regulates the pro‐angiogenic functions of macrophages through the PI3K‐Akt signaling pathway, we utilized LY294002, an inhibitor of the PI3K‐Akt signaling pathway.^[^ ^42^, ^43^, ^44^, ^45^ ^]^ Specifically, when BMDMs were cultured on BTNF nanocomposite membranes, we added LY294002 at the same time. After stimulating with LY294002 for 24 h, the medium was discarded and replaced with fresh complete medium, and the cells were further cultured for 48 h. The conditioned medium was then collected for subsequent experiments. Following this, HUVECs were cultured in the collected conditioned medium for 4 h (Figure 5B). The results of the 2D tube‐formation assay showed that the addition of LY294002 significantly inhibited the tube‐formation capacity of HUVECs (Figure 5C,D). Additionally, we also observed the same results from RT‐qPCR and western blot analyses whereby the addition of LY294002 significantly decreased the gene expression levels of *CD31* and *VEGF* and the protein expression levels of CD31 and CD34 (Figure 5E–G). In summary, these results indicated that HEPD induced the pro‐angiogenic polarization of macrophages by activating the PI3K‐Akt signaling pathway, which promoted angiogenesis (Figure 5H), ultimately enhancing bone defect repair.

## 3 Conclusion

In this study, we elucidated how electrical signals at the micro‐ and nanoscale regulate the immune response to promote bone regeneration. We found that, when compared to the HOPD, HEPD could significantly better promote the polarization of macrophages toward the M2 phenotype. This transition endowed the immune microenvironment with anti‐inflammatory properties during the initial stages of bone defect repair, which was beneficial to subsequent tissue regeneration. Specifically, the HEPD activated the PI3K‐Akt signaling pathway in macrophages to enhance their pro‐angiogenic functions, thereby enabling them to promote subsequent angiogenesis of endothelial cells. Ultimately, this cascade of biological processes accelerated the bone defect repair process. Hence, this study provides novel insights into the in‐depth understanding of the essence of micro‐ and nanoscale electrical signal‐mediated regulation of tissue regeneration. Additionally, cues for enhancing the therapeutic efficacy of electroactive biomaterials were also provided. Moreover, the focus of this study is on macrophages. Following this cue, future research should prioritize studies on how heterogeneous electrical signals at the micro‐ and nanoscale regulate the interaction between macrophages with other immune cells, as well as with non‐immune cells to promote tissue regeneration. This will contribute to a more comprehensive and accurate understanding of the mechanisms of action of these heterogeneous electrical signals, thereby providing a theoretical basis for precise improvement of the properties of electroactive biomaterials and ensuring the safety of their clinical applications in the future.

## 4 Experimental Section

### Fabrication of BaTiO_3_ Nanofibers (BTNF) and BaTiO_3_ Nanoparticles (BTNP) Nanocomposite Membranes

The BTNF was fabricated as described in previous study.^[^ ^10^ ^]^ Barium acetate, tetrabutyl titanate, and acetylacetone (TONG GUANG, China) were dissolved in acetic acid (Macklin, China) at a 1:1:2 molar ratio and stirred to yield a homogeneous barium titanate precursor solution. The viscosity of the solution was adjusted by adding polyvinylpyrrolidone (PVP, M = 1 300 000) (Aladdin, China) to the precursor solution. The mixed solution was then subjected to electrospinning under an applied electric field of 1.5 kV cm^−1^. BTNF was obtained by calcining electrospun fibers consisting of PVP and barium titanate precursor at 750 °C for 6 h. The BTNF was dispersed by ultrasonication for 5 min. BTNP with a diameter of 100 nm was procured from Sigma–Aldrich Inc. (St. Louis, USA). Subsequently, BTNF and BTNP were surface‐modified based on the protocol of the previous studies.^[^ ^2^, ^10^ ^]^ Briefly, BTNF and BTNP were mixed with a 0.01 mol L^−1^ dopamine hydrochloride (Alfa Aesar, USA) solution and stirred for 12 h at 60 °C. Finally, the dopamine‐modified BTNF or BTNP was incorporated into P(VDF‐TrFE) (70/30 mol% VDF/TrFE) (Arkema, France) co‐polymer powders dissolved in N,N‐dimethylformamide (DMF) (TONG GUANG, China) at a polymer matrix concentration of 5 vol%. The mixture was stirred overnight to form a stable suspension and then cast as a membrane on a Quartz glass substrate. The membranes were dried at 55 °C for 10 h to volatilize the DMF. Importantly, the membranes were polarized by subjecting them to a corona discharge at room temperature under a 15 kV DC field for 30 min.

### Characterization of the BTNF and BTNP Nanocomposite Membranes

The surface morphology of the nanocomposite membranes after polarization was characterized using SEM JSM‐7001F (JEOL, Japan). The surface potential and roughness were examined with SPM using a Bruker Dimension Icon instrument (Bruker, USA). The piezoelectric coefficient *d_33_* was determined by a Quasistatic *d_33_* instrument, ZJ‐ 6A (JKZC, China).^[^ ^46^ ^]^ Phase structures were analyzed via X‐ray diffraction spectroscopy using a Rigaku D/max 2500VB2t/PC (Rigaku, Japan). The P‐E loop was assessed using a Ferroelectric tester, Precision 10 kV HVI‐SC (Radiant Technologies, USA).

### Animals and Surgical Procedures

The cranial bone defect model was established using 6‐week‐old male C57BL/6 mice in this study. All animal experiments were conducted under the approval of the Animal Care and Use Committee of Peking University (IACUC No. LA2022682). The mice were anesthetized with 50 mg kg^−1^ of pentobarbital sodium via intraperitoneal injections, followed by the exposure of the dorsal cranium. Two full‐thickness bone defects, each 2 mm in diameter, were created at the center of each mouse's parietal bone using a saline‐cooled trephine drill. The bone defects were covered with either polarized BTNF nanocomposite membranes or polarized BTNP nanocomposite membranes. The whole calvarias were collected for evaluation at multiple time points: 3 days, 7 days, 2 weeks, and 12 weeks post‐surgery.

To deplete macrophages, 6‐week‐old male C57BL/6 mice weighing ≈20 g were administered clodronate liposome (Yeasen Biotechnology, China) at a dosage of 200 µL per mouse, via intravenous injection, at 48 h before surgery. Injections were administered every 3 days until the 7th day post‐surgery. The control group received the same dose of control liposomes (Yeasen Biotechnology, China).^[^ ^38^, ^39^, ^40^ ^]^

### Cell Culture

BMDMs were cultured in dulbecco's modified eagle medium (Procell, China) supplemented with 10% (v v^−1^) fetal bovine serum (Procell) and 1% (v v^−1^) penicillin/streptomycin solution (Procell). HUVECs (Oricell, China) were cultured in endothelial cell medium (Oricell). All cells were cultured within a humidified incubator containing 5% CO_2_ at 37 °C. LY294002 (MedChemexpress, USA) at 10 μм concentration was used as an inhibitor of the Pl3K‐Akt signaling pathway.^[^ ^42^, ^43^, ^44^ ^]^

### Flow Cytometry

BMDMs treated with different nanocomposite membranes were collected, fixed, and permeabilized using 100% methanol. Subsequently, the cells were incubated with primary antibodies at 4 °C for 30 min. The primary antibodies used were as follows: CD86 Polyclonal antibody (1:500; Proteintech, USA) and APC‐CD206 (0.5 µg per million cells in 100 µL volume; Biolegend, USA). Following incubation with the primary antibody, the cells were washed with phosphate‐buffered saline (PBS) (Solarbio, China) and incubated with the secondary antibody Goat Anti‐Rabbit IgG H&L (Alexa Fluor 488) (Abcam, UK) at 4 °C for 30 min. Analysis was conducted using LSRFortessa flow cytometers (BD Biosciences, USA), and data were processed using FlowJo software (Tree Star, USA).

### RT‐qPCR

RNA from BMDMs or HUVECs was extracted using Trizol reagent (Invitrogen, USA). The concentration of RNA was measured at 260 nm using a NanoDrop ND‐2000 spectrophotometer (Thermo Fisher Scientific, USA). Reverse transcription was performed using a PCR thermal cycler (Takara, Japan). PCR was performed on a 96‐well optical reaction plate with a total volume of 20 µL, comprising 1 µL of template cDNA, 10 µL of FastStart Universal SYBR Green Master Mix, 8 µL of RNase‐free water, and 1 µL of primer. PCR amplification was conducted with the following cycling parameters: 15 min at 95 °C, followed by 45 cycles of 15 s at 95 °C and 1 h at 60 °C. Differences in gene expression levels between the groups were statistically analyzed after processing the raw data. All reactions were conducted in triplicates. Glyceraldehyde‐3‐phosphate dehydrogenase (*GAPDH*) was used as the internal control. The primer sequences are listed in Table S1 (Supporting Information).

### Western Blot

Cells were lysed using radioimmunoprecipitation assay buffer (Beyotime, China) containing a protease and phosphatase inhibitor cocktail (Thermo Fisher Scientific), followed by centrifugation at 12 000 rpm for 20 min at 4 °C to collect the supernatants. The protein concentration was determined using a bicinchoninic acid protein assay kit (Beyotime). Samples were mixed with 6 × sodium dodecyl sulfate loading buffer (Beyotime) at a 1:5 ratio and heated at 100 °C for 5 min to denature the proteins. Twenty micrograms of protein were separated by sodium dodecyl sulfate polyacrylamide gel electrophoresis and transferred onto a polyvinylidene fluoride membrane. The membranes were blocked with 5% (w v^−1^) skimmed milk (diluted with tris buffered saline with tween 20) for 1 h at room temperature, followed by overnight incubation with the primary antibody at 4 °C, and subsequent incubation with an horseradish peroxidase (HRP)‐conjugated secondary antibody for 1 h at room temperature. Autoradiograms were obtained using Enhanced Chemiluminescence Western Blotting Substrate (Abclonal, China). GAPDH served as the internal control. The primary antibodies used were as follows: Anti‐GAPDH antibody (1:2000; Abcam), Anti‐Mannose Receptor (CD206) antibody (1:1000; Abcam), CD86 Polyclonal antibody (1:1000; Proteintech), Anti‐CD31 antibody (1:1000; Abcam), and CD34 Polyclonal antibody (1:1000; Proteintech). The HRP‐conjugated secondary antibodies used were as follows: HRP‐labeled Goat Anti‐Rabbit IgG(H + L) (1:1000; Beyotime) and HRP‐labeled Goat Anti‐Mouse IgG(H + L) (1:1000; Beyotime).

### Immunohistochemical Staining

For nanocomposite membranes and BMDMs, the samples were rinsed with PBS and fixed with 4% (w v^−1^) paraformaldehyde solution (Solarbio) for 30 min at room temperature. After permeabilizing with 0.1% (w v^−1^) Triton X‐100 (Solarbio) in PBS for 10 min, the samples were blocked with 3% bovine serum albumin (BSA) (Solarbio) in PBS for 1 h at room temperature. After washing with PBS, the samples were incubated with primary antibodies in 5% (w v^−1^) BSA in PBS overnight at 4 °C. After rinsing to remove excess antibodies, the samples were incubated with secondary antibodies in the dark for 1 h at room temperature. The secondary antibody used was goat anti‐rabbit IgG H&L (Alexa Fluor 488) (1:1000; Abcam). Phalloidin (1:250; Solarbio) was utilized for staining the cytoskeleton, and 4′,6‐diamidino‐2‐phenylindole (DAPI; 1:500; Solarbio) was employed for cell nuclei staining, both in the dark for 30 min at room temperature.

For calvaria tissues, the samples were fixed with 4% (w v^−1^) paraformaldehyde solution (Solarbio) for 24 h, and then decalcified and dehydrated according to standard protocols, paraffin‐embedded, and sectioned at a thickness of 5 µm. The samples were deparaffinized, dehydrated, and subjected to antigen retrieval. Then, they were permeabilized with 0.1% (w v^−1^) Triton X‐100 (Solarbio) in PBS for 10 min, blocked with 3% (w v^−1^) BSA (Solarbio) in PBS for 30 min, and then incubated with primary antibodies overnight at 4 °C. After washing with PBS, the samples were incubated with secondary antibody for 30 min at room temperature. After another round of antigen retrieval treatment, the next antibody was stained in the same manner. The cell nuclei were stained with DAPI (Solarbio).

Images were captured with a TCS‐SP8 STED 3X confocal laser scanning microscope (Leica, Germany). The primary antibodies used were as follows: Anti‐Mannose Receptor (CD206) antibody (1:1000; Abcam), CD86 Polyclonal antibody (1:500; Proteintech), Alexa Fluor 488 Anti‐CD68 antibody (1:250; Abcam), Anti‐CD31 antibody (1:1000; Abcam), OCN Polyclonal antibody (1:500; Proteintech), and Anti‐IL‐10 antibody (1:500; Abcam).

### Tube‐Formation Assay

24‐well plates were coated with 250 µL of Matrigel (BD Biosciences) per well, ensuring that no air bubbles were introduced. The plates were initially placed in a refrigerator at 4 °C for 10 min to level the Matrigel, then transferred to an incubator for at least 30 min to solidify the Matrigel. After incubating at 37 °C in a 5% CO_2_ incubator for 4 h, the tubular structures were observed under confocal microscopy. The Image Pro Plus software was used to evaluate the tube formation.

### Sample Collection for Evaluation of Vascular Formation and Microvascular Perfusion

After anesthesia, mice were successively perfused with heparinized saline, 4% (w v^−1^) paraformaldehyde solution, and microfil (Flow Tech, USA)^[^ ^47^, ^48^ ^]^ through the cardiac artery. The culled mice were then stored overnight at 4 °C. Subsequently, calvarial specimens were collected and fixed in 4% (w v^−1^) paraformaldehyde solution (Solarbio) for 24 h.

### Micro‐CT Scanning Analysis

The mouse calvaria samples were fixed in 4% (w v^−1^) paraformaldehyde solution (Solarbio) at 4 °C for 24 h and subsequently scanned using a micro‐CT SkyScan 1276 instrument (Bruker). The vascular microfil‐perfusion calvaria samples were scanned after decalcification. 3D reconstruction was performed using the accompanying NRecon software, and 3D visualization was achieved using Data Viewer and CTvox software, with the volume of regenerated bone or vascular network being quantitatively analyzed using the CTAn software.

### Bulk RNA‐seq

RNA was extracted from BMDMs using standard methods. RNA integrity was evaluated using the RNA Nano 6000 Assay Kit on the Bioanalyzer 2100 system (Agilent Technologies, USA). To target cDNA fragments between 370 and 420 bp in length, library fragments were purified using the AMPure XP system (Beckman Coulter, USA). PCR products were purified with the same system, and library quality was evaluated with the Agilent Bioanalyzer 2100 system. The index‐coded samples were clustered using the cBot Cluster Generation System with the TruSeq PE Cluster Kit v3‐cBot‐HS (Illumina, USA) following the manufacturer's instructions. After cluster generation, the library preparations were sequenced on an Illumina Novaseq platform, producing 150 bp paired‐end reads. Differential expression analysis between the two groups was performed using the DESeq2 R package (1.20.0). GO enrichment analysis of differentially expressed genes was implemented by the clusterProfiler R package, in which gene length bias was corrected. ClusterProfiler R package was also used to test the statistical enrichment of differentially expressed genes in the Kyoto Encyclopedia of Genes and Genomes pathways.

### Statistical Analysis

Statistical analyses were performed using GraphPad Prism (version 9.0) software (USA). All quantitative data were expressed as mean ± standard deviation (SD) of at least three independent replicate experiments. The Shapiro‐Wilk test was used to assess the normality of distributions. Statistical differences were analyzed using the unpaired Student's t‐test for independent samples. Statistical significance levels were set at *p < 0.05, **p < 0.01, ***p < 0.001, and ****p < 0.0001. A *p*‐value of less than 0.05 was considered statistically significant.

## Conflict of Interest

The authors declare no conflict of interest.

## Supporting information



Supporting Information

## Data Availability

The data that support the findings of this study are available from the corresponding author upon reasonable request.
