# Supplementary material for: Manipulation of Surface Potential Distribution Enhances Osteogenesis by Promoting Pro‐Angiogenic Macrophage Polarization via Activation of the PI3K‐Akt Signaling Pathway
Source: Adv Sci (Weinh). 2024 Dec 30;12(8):2414278. doi: 10.1002/advs.202414278 (PMC11848552; doi:10.1002/advs.202414278)
Supplement: Supplementary file 1 — Supporting Information [file ADVS-12-2414278-s001.docx]

Supporting Information

**Manipulation of Surface Potential Distribution Enhances Osteogenesis by Promoting Pro-angiogenic Macrophage Polarization via Activation of the PI3K-Akt Signaling Pathway**

*Qun Cui, Xiaona Zheng, Yunyang Bai, Yaru Guo, Shuo Liu, Yanhui Lu, Lulu Liu, Jia Song, Yang Liu, Boon Chin Heng, Fuping You*, Mingming Xu*, Xuliang Deng, Xuehui Zhang**


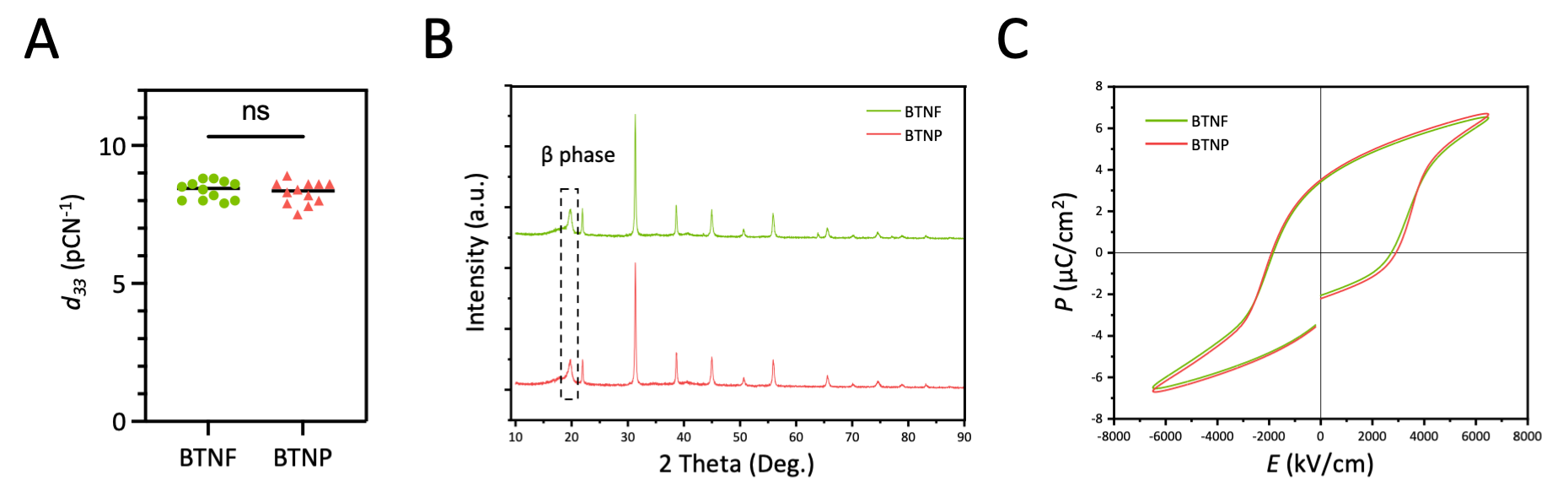


**Figure S1. Characterization of the electrical properties of the BTNF and BTNP nanocomposite membranes.**

(A) Piezoelectric constants *d_33_* of BNTF and BTNP nanocomposite membranes, *n*=12. (B) XRD patterns of BNTF and BTNP nanocomposite membranes. Peaks of the polar β‐phase were marked with a frame. (C) Hysteresis loops of BNTF and BTNP nanocomposite membranes. Statistical significance was assessed using the unpaired Student’s t-test. ns, not significant.

**Table S1. Primer sequences used in this study.**

| Target gene | Forward sequence (5′-3′) | Reward sequence (5′-3′) |
| --- | --- | --- |
| *Gapdh* | AGTATGACTCCACTCACGGC | AGACACCAGTAGACTCCACG |
| *Cd86* | GCCACCCACAGGATCAATTA | GTGCAGGTCAAATTTATGCC |
| *Cd206* | ATGAACAAGCATTCCTGACTAG | GTCGGCATTCCAGTGTGTAAAC |
| *Arg1* | ACACTGACATCAACACTCCC | ACACGATGTCTTTGGCAGAT |
| *Tnfα* | GCGACGTGGAACTGGCAGA | CCGATCACCCCGAAGTTCA |
| *GAPDH* | TCAAGAAGGTGGTGAAGCAGG | TCAAAGGTGGAGGAGTGGGT |
| *CD31* | TGACCCTTCTGCTCTGTTCAA | CTGAGGCTTGACGTGAGAGG |
| *VEGF* | GGAGGAGGGCAGAATCATCA | CTTGGTGAGGTTTGATCCGC |
